# Tissue Derivation and Biological Sex Uniquely Mediate Endothelial Cell Protein Expression, Redox Status, and Nitric Oxide Synthesis

**DOI:** 10.3390/cells12010093

**Published:** 2022-12-26

**Authors:** Rami S. Najjar, Brett J. Wong, Rafaela G. Feresin

**Affiliations:** 1 Department of Nutrition, Georgia State University, Atlanta, GA 30303, USA; 2 Department of Kinesiology & Health, Georgia State University, Atlanta, GA 30303, USA

**Keywords:** cardiovascular disease, endothelial function, nitric oxide, sex hormones, angiotensin, oxidative stress, inflammation, human aortic endothelia cells, human umbilical vein endothelial cells, human microvascular endothelial cells

## Abstract

Human endothelial cells are routinely utilized in cardiovascular research to provide a translational foundation for understanding how the vascular endothelium functions in vivo. However, little attention has been given to whether there are sex specific responses in vitro. Similarly, it is unclear whether endothelial cells derived from distinct tissues behave in a homogenous manner. Herein, we demonstrate that marked sex differences exist within, and between, commonly utilized human primary endothelial cells from healthy donors, with respect to redox status, nitric oxide synthesis, and associated proteins that can mediate their expression. Further, we demonstrate that endothelial cells respond uniquely to inflammatory insult in a sex- and tissue origin-dependent manner. Our findings suggest sex and tissue derivation may need to be considered when studying endothelial cells in vitro as cells derived from distinct tissue and sexes may not behave interchangeably.

## 1. Introduction

In vitro investigations are a cornerstone of cardiovascular research and play a key role in the development of pharmacological therapies and furthering understanding of vascular biology. Endothelial cells are critically involved in the development of atherosclerosis and hypertension, and thus, have been instrumental in progressing our understanding of these diseases. However, substantial heterogeneity exists regarding primary cell lines selected by investigators to study these diseases. Human umbilical vein endothelial cells (HUVECs) are most frequently used due to their ease in procurement, while human aortic endothelial cells (HAECs) and human microvascular endothelial cells (HMVECs) are also used but to a lesser extent. These cells are often used interchangeably, but they may not be physiologically relevant in all vascular diseases. For example, studying the effect of oxidized low-density lipoproteins in the progression of atherosclerosis using HUVECs as a proxy for the endothelium of the coronary artery despite HUVECs playing no physiological role in atherosclerosis in vivo [1]. 

In addition, little attention has been given to the sex of the cell donor. Biological sex is significant considering that cardiovascular disease (CVD) incidence between sexes is not equal. For example, men have a higher incidence of coronary artery disease (CAD), [2] and hypertension (51%) compared to women (39.7%) [3]. In contrast, ischemia with non-obstructive CAD, primarily driven by coronary microvascular dysfunction, is more prevalent in women than in men [4]. 

Endothelial function is usually assessed as nitric oxide (NO)-dependent vasodilation and is predictive of CVD. As such, endothelial dysfunction and reduced NO-dependent vasodilation are viewed as a non-traditional risk factor for CVD. NO derived from endothelial NO synthase (eNOS) are critically involved in vasodilation, and proper eNOS function is classically associated with a healthy endothelium [5]. Development of oxidative stress and inflammation is multifactorial but pathological components of the renin angiotensin (Ang) system (RAS) have been shown to be a major contributor. For example, binding of Ang II to the Ang II type 1 receptor (AT_1_R) leads to pathological effects in the endothelium due to oxidative stress derived from both NADPH oxidases (NOX) and mitochondria [6,7], and inflammatory signaling via nuclear factor kappa-light-chain-enhancer of activated B cells (NF-κB) and mitogen activated protein kinases (MAPKs) [8]. These consequences generally drive endothelial dysfunction, promoting vasoconstriction and atherosclerosis development [9]. In healthy conditions, endogenous antioxidant enzymes neutralize reactive oxygen species (ROS).

Although reduced NO-dependent vasodilation is the predominant phenotype of endothelial dysfunction, mechanisms leading to reduced bioavailable NO and endothelial dysfunction are not fully understood. In humans, men tend to have higher systemic oxidative stress than women, suggesting sex-dependent differences in downstream cellular mechanisms may contribute to differences in CVD incidence [10]. In addition, estrogen receptors (ERs) have been closely tied to attenuation of hypertension [11,12,13], endothelial dysfunction [12,13,14] and atheroma formation [15,16]. In contrast, the effects of androgen receptors (AR) are equivocal [17]. For example, AR stimulation increased eNOS activity in HAECs [18], while inflammation via NF-κB was upregulated in HUVECs [19]. Collectively, these data suggest mechanisms of endothelial dysfunction may differ depending on which tissue cells are derived from and/or by biological sex of the cell donor. To our knowledge, a comprehensive study assessing differences in tissue derivation and sex contributions to mechanisms involved in endothelial function has yet to be performed. 

Therefore, the aim of this study was to investigate differences between sex and cell derivation in three human, primary endothelial cell types: HUVECs, HAECs and dermal HMVECs. To gain additional insights and enhance physiological relevance under simulated CVD conditions, we also assessed whether these cells behaved differently under inflammatory insult such as tumor necrosis factor (TNF)-α. We initially hypothesized that we would observe similar sex differences regardless of tissue derivation. On the contrary, our findings demonstrate intrinsic differences due to both cell origin and biological sex. 

## 2. Materials and Methods

### 2.1. Reagents

All reagents used are listed in Supplementary Table S1.

### 2.2. Cell Culture

Primary, single donor HAECs (Cat #: 304-05a) from a 31-year-old Caucasian male (Lot #: 3118) and a 20-year-old Caucasian female (Lot #: 2887), HUVECs (Cat #: 200-05n) from a neonate Caucasian male (Lot #: 3451) and a neonate Caucasian female (Lot #: 3455) and HMVECs (Cat #: 100-05a) from a 49-year-old Caucasian male (Lot #: 2092) and 40-year-old Caucasian female (Lot #: 1710) were purchased from Cell Applications (San Diego, CA, USA). All donors were considered healthy. HAECs and HUVECs were cultured in complete endothelial cell medium (basal medium + supplement) and HMVECs were cultured in complete HMVEC medium. Upon reaching 80% confluency, cells were detached with Accutase^®^, centrifuged at 220× *g* for 5 min, then seeded in 60 mm dishes, 24-well plates, or clear bottom 96-well black plates at a density of 10,000 cm^2^. Upon reaching 80% confluency, cells were starved (0.5% FBS) and treated with or without 20 ng/mL TNF-α. After 24 h, cells were used for downstream applications. HUVECs and HMVECs were utilized <15 population doublings while HAECs were utilized <12 population doublings as per manufacturer instructions. Population doubling refers to the number of times the cell population underwent mitosis to duplicate. All cells were utilized within 6-8 passages, which was under the population doubling limit.

### 2.3. Western Blot

Cells were collected with RIPA buffer supplemented with phosphatase inhibitor cocktail 1 and 2 and protease inhibitor cocktail followed by centrifugation at 16,000× *g* for 20 min at 4 °C. Protein from supernatant was quantified and normalized via the DC protein assay kit (BioRad Laboratories, Hercules, CA, USA). In preparation for sodium dodecyl sulfate (SDS) polyacrylamide gel electrophoresis, 20 μg of samples were mixed with 4 × Laemmli buffer and 10% 2-mercaptoethanol (BioRad Laboratories, Hercules, CA, USA). Samples were then briefly vortexed, centrifuged, then heated for 10 min at 70 °C in a dry heating block and loaded onto a polyacrylamide gel for electrophoresis. Following electrophoresis, gels were transferred to a polyvinylidene difluoride (PVDF) membrane using a Trans-Blot Turbo Transfer System (Bio-Rad Laboratories, Hercules, CA, USA). Membranes were then blocked in TBS-T (50 m mol/L Tris, 150 m mol/L NaCl, 0.2% Tween-20, pH 7.4) + 5% non-fat dry milk (NFDM) and washed in TBS-T (3 × 5 min).

Membranes were incubated overnight in 4 °C with primary antibody diluents (1:1000 dilution) containing TBS-T + 5% bovine serum albumin (BSA). Membranes were then washed in TBS-T (3 × 5 min) and probed with secondary antibodies (1:5000 dilution) in TBS-T + 5% NFDM for 1 h at room temperature. After three 5 min washes with TBS-T, chemiluminescent imaging of target proteins was performed with Horseradish peroxidase (HRP) substrate using the ChemiDoc Imaging Systems (BioRad Laboratories, Hercules, CA, USA). Band density of protein of interest was quantified using Image Lab 6.0 (BioRad Laboratories, Hercules, CA, USA) and normalized to the loading control protein (β-actin or GAPDH). With respect to phosphorylated proteins and their total protein controls (i.e., eNOS, p65, and Akt), if both were on the same membrane, the phosphorylated protein was normalized to the respective total control. However, if phosphorylated and total proteins were on different membranes, they were normalized to their respective loading control (β-actin or GAPDH), followed by their respective total control.

### 2.4. mRNA Analysis

RNA was extracted from the left ventricle of the heart using TRI reagent. RNA was quantified using the Nanodrop ND 1000 Spectrophotometer. 2 µg RNA was reverse transcribed to produce cDNA. Gene expression was measured by real-time PCR using a LightCycler 96 (Roche, Penzberg, Germany) by measurement of SYBR Green. mRNA levels were normalized to cyclophilin expression and were analyzed using the 2−ΔΔCT method. Cyclophilin expression levels were unchanged in response to diet or treatment group. Primer sequences are displayed in Supplementary Table S2.

### 2.5. Measurement of ROS

In a black, clear-bottom 96-well plate following 24 h treatments in starvation medium, H_2_DCFDA or DHE dissolved in dimethyl sulfoxide were added to wells (10 µM final concentration) followed by 30 min incubation (37 °C and 5% CO_2_). Cells were then gently washed with warm phosphate-buffered saline (PBS) twice, and phenol red-free starvation medium supplemented with NucBlue™ (1 drop/mL) was added. Fluorescence was read using a microplate reader at the following Ex/Em (nm): 495/527 (H_2_DCFDA), 518/606 (DHE; O_2_^−^) 480/576 (DHE; non-specific radicals) and 360/460 (Hoechst 33342 with NucBlue™). Fluorometric values for H_2_DCFDA and DHE were normalized to Hoechst 33342 to account for any differences in cell density.

### 2.6. Immunocytochemistry

Poly-L-lysine cover slips (#1.5 thick) were placed in 24-well plates and incubated with gelatin containing solution (attachment factor solution, Cell Applications) for additional matrix. Following 24 h treatments, cells were fixed in media with 2% paraformaldehyde (PFA) for 2 min, followed by 18 min in 2% PFA in PBS. Wells were washed with PBS and incubated with a 0.5% Triton solution for 5 min to allow for permeabilization. Wells were then rinsed and blocked in a solution containing 5% BSA in PBS with 0.3% Triton (PBS-T) for 1 h. Following wash with PBS-T, cells were incubated with a PBS-T diluent containing 1% BSA + nitrotyrosine primary antibody (dilution 1:400) or a diluent without antibody to serve as a blank control. Following overnight incubation at 4 °C in a humidified dark incubator, wells were washed with PBS-T, followed by incubation with secondary antibody (1:1000) in 1% BSA in PBS-T for 1 h in the dark at room temperature. Wells were washed (3×) then a 3,3′-diaminobenzidine (DAB) chromogen concentrate/DAB diluent solution were added to each well for 5 min. Cells were counterstained with Mayer’s hematoxylin solution and dehydrated in two changes of 95% and 100% ethanol. Cover slips were then mounted on slides for visualization. Individual cells were quantified with Image J version 1.53 (NIH, Bethesda, MD, USA).

### 2.7. Intracellular NO

Following 24 h treatment, DAF-2 DA was added to each well at a final concentration of 5 µM. After a 30 min incubation in 37 °C and 5% CO_2_, cells were washed twice in warm PBS, then placed in warm phenol red-free medium for immediate imaging at a fluorescent intensity of 485/528 nm (Ex/Em). To account for variation in cell density between wells and to avoid the detection of apoptotic cells which fluoresce intensely, individual cells of normal spindle structure were quantified with Image J version 1.53 (NIH, Bethesda, MD, USA).

### 2.8. Statistical Analysis

GraphPad Prism version 9.3 (San Diego, CA, USA) was used for all statistical analyses. Data were assessed for normality using Shapiro–Wilk test. Data were statistically analyzed with one-way ANOVA followed by Tukey post hoc multiple comparison analysis. Data are expressed as mean ± standard deviation of the mean (SD). Statistical significance was set a priori at *p* ≤ 0.05.

## 3. Results

### 3.1. Sex Receptor Expression

**Figure 1:**
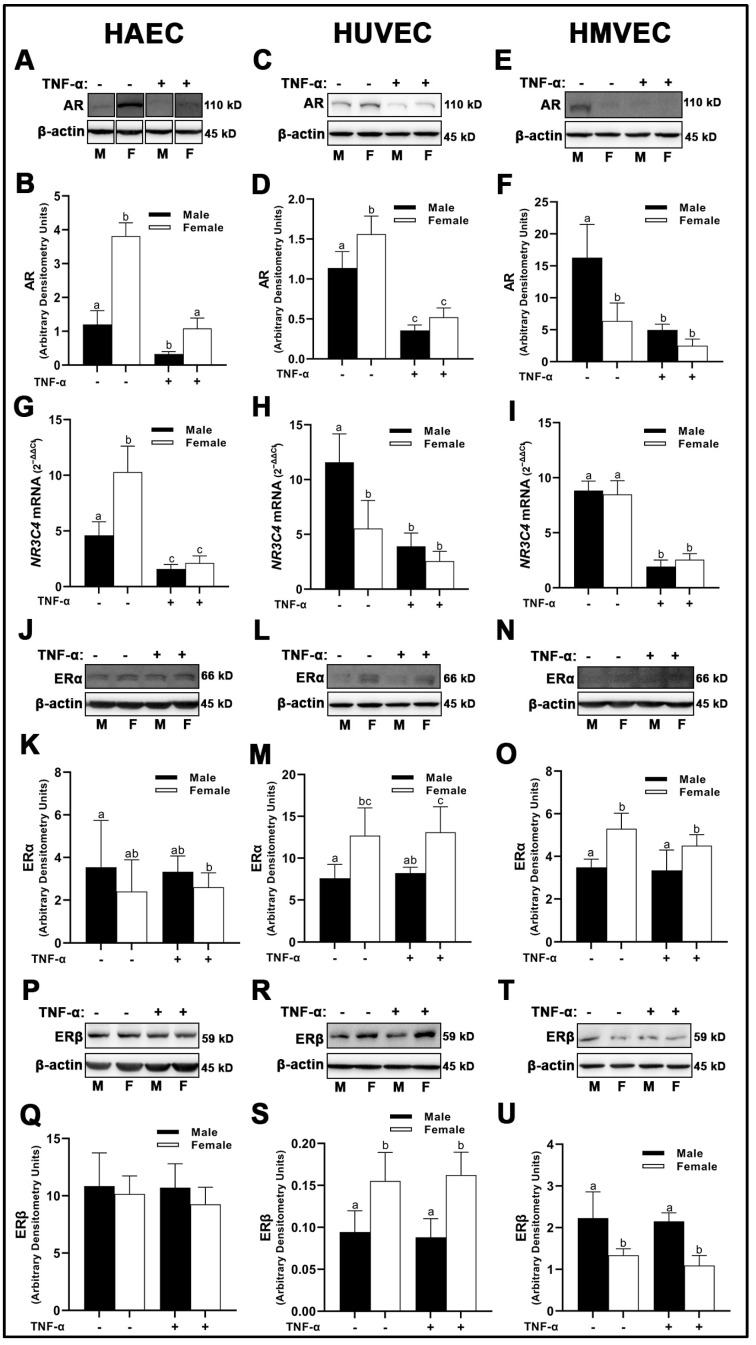
Expression of sex receptors in male and female endothelial cells. HAECs, HUVECs and HMVECs were cultured in starvation medium at ~80% confluency for 24 h with or without TNF-α (20 ng/mL). Protein expression of AR (**A**–**F**), ERα (**J**–**O**), and ERβ (**P**–**U**) were determined by Western blot. Quantification was performed using Image Lab (Bio-Rad Laboratories, Inc.). Protein was normalized to respective control bands (β-actin). Gene expression of AR (**G**–**I**) were determined with qPCR. Genes of interest were normalized to housekeeping gene cyclophilin. Data are expressed as mean ± SD from 3–6 experiments. Data were statistically analyzed with one-way ANOVA followed by Tukey post hoc multiple comparison analysis between groups. Values that do not share the same letter are significantly different from each other (*p* ≤ 0.05).

The protein expression of AR was significantly greater in females compared to males in both HAECs (Figure 1A,B) and HUVECs (Figure 1C,D); however, male HMVECs had greater AR compared to females (Figure 1E,F). Due to the unusually increased expression of AR in females compared to males in both HAECs and HUVECs, we assessed *NR3C4* mRNA expression to confirm these findings. *NR3C4* mRNA reflected protein expression between male and female HAECs (Figure 1G), but not HUVECs (Figure 1H) or HMVECs (Figure 1I) suggesting translation was not fully reflective of mRNA concentrations. Inflammatory insult with TNF-α substantially reduced expression of AR at the transcriptional and protein level for all cell lines, and molecular weights of protein bands were validated in positive control lysates derived from LNCaP and MCF-7 cells (data not shown), suggesting that the protein expression was reflective of true AR density. ERα were weakly expressed across all three cell lines via Western blot, and molecular weights were confirmed with MCF-7 control lysate run in parallel (data not shown). Nonetheless, HUVECs and HMVECs expressed greater ERα in basal female cells compared to male cells with little effects of TNF-α on ERα expression (Figure 1L–O). In contrast, there were no statistically significant differences in ERα expression between male- and female-derived HAECs irrespective of treatment (Figure 1J–K). ERβ was expressed differently across each cell line with male HMVECs expressing greater levels than female-derived cells (Figure 1T,U), female HUVECs had greater expression levels than male-derived cells (Figure 1R,S), and no statistical differences between males and females were noted in HAECs irrespective of inflammatory insult with TNF-α (Figure 1P,Q). In summary, these data illustrate that sex is not a determining factor in sex receptor expression, as divergent effects were observed among the three human primary endothelial cells evaluated. Further, we demonstrate that inflammatory insult with TNF-α reduces AR expression irrespective of sex with no effect on ERα or ERβ.

### 3.2. Renin-Angiotensin System (RAS) Components

**Figure 2:**
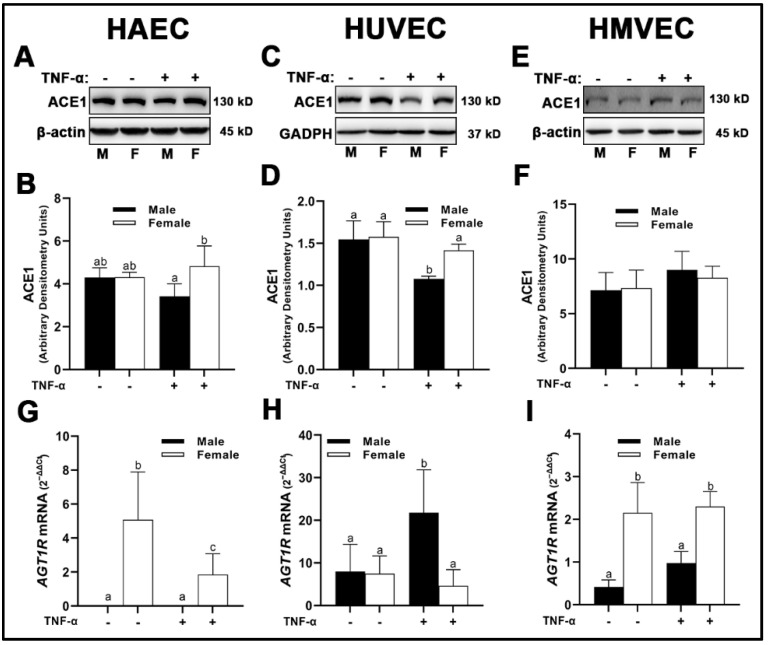
Expression of pathological RAS components in male and female endothelial cells. HAECs, HUVECs and HMVECs were cultured in starvation medium at ~80% confluency for 24 h with or without TNF-α (20 ng/mL). Protein expression of ACE1 (**A**–F) were determined by Western blot. Quantification was performed using Image Lab (Bio-Rad Laboratories, Inc.). Protein was normalized to respective control bands (β-actin or GAPDH). Gene expression of AT_1_R (**G**–**I**) were determined with qPCR. Genes of interest were normalized to housekeeping gene cyclophilin. Data are expressed as mean ± SD from 3–6 experiments. Data were statistically analyzed with one-way ANOVA followed by Tukey post hoc multiple comparison analysis between groups. Values that do not share the same letter are significantly different from each other (*p* ≤ 0.05).

We observed similar expression of angiotensin converting enzyme (ACE)1 between sexes across all three human primary endothelial cells under basal conditions. Divergent effects were observed upon inflammatory insult with TNF-α. While HMVECs were relatively unaffected (Figure 2E,F) with TNF-α stimulation, male HAECs treated with TNF-α had reduced ACE1 expression compared with female HAECs (Figure 2A,B); however, neither were statistically significant different from basal levels. In contrast, male HUVECs treated with TNF-α expressed reduced ACE1 which was significantly lower than male and female cells under basal conditions as well as female cells treated with TNF-α (Figure 2C,D). In male-derived HAECs, we were unable to detect mRNA expression of *AGT1R* (Figure 2G). Female-derived HAECs did express *AGT1R* mRNA, with greater expression observed in cells grown under basal conditions compared with TNF-α insult. Similar sex-dependent differences were observed in HMVECs, with significantly greater *AGT1R* mRNA gene expression in female- compared with male-derived cells (Figure 2I), albeit *AGT1R* mRNA was detectable in male-derived HMVECs unlike male-derived HAECs. In contrast, *AGT1R* mRNA was significantly greater in male-derived HUVECs treated with TNF-α compared with basal male cells and female cells irrespective of treatment (Figure 2H).

**Figure 3:**
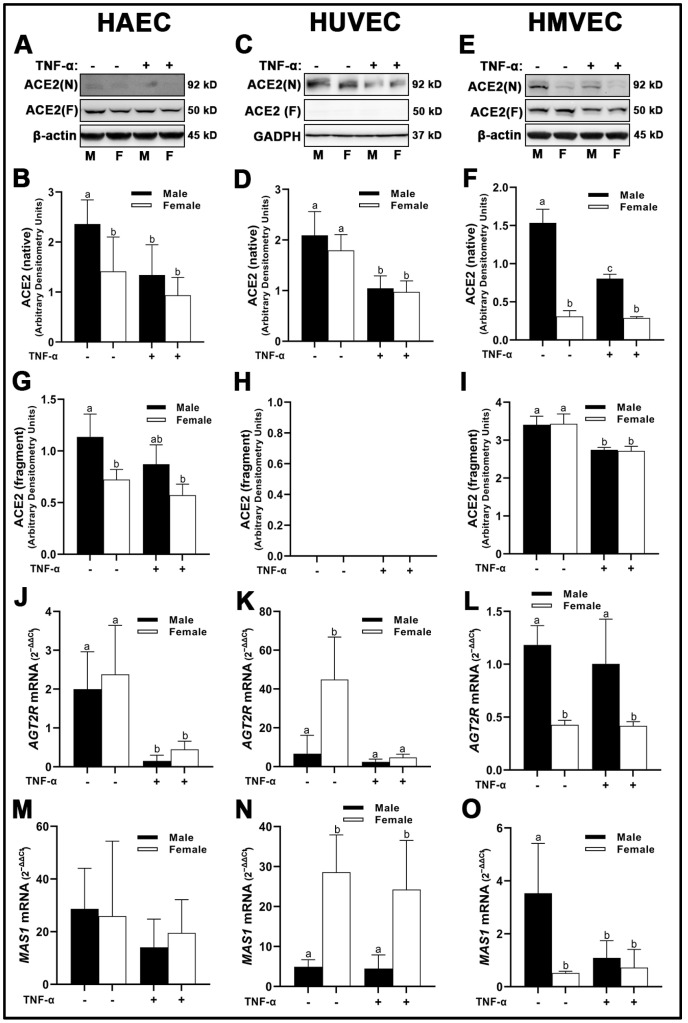
Expression of protective RAS components in male and female endothelial cells. HAECs, HUVECs and HMVECs were cultured in starvation medium at ~80% confluency for 24 h with or without TNF-α (20 ng/mL). Protein expression of ACE2 (**A**–**I**) were determined by Western blot. Quantification was performed using Image Lab (Bio-Rad Laboratories, Inc.). Protein was normalized to respective control bands (β-actin or GAPDH). Gene expression of AT_2_R (**J**–**L**) and Mas receptor (**M**–**O**) were determined with qPCR. Genes of interest were normalized to housekeeping gene cyclophilin. Data are expressed as mean ± SD from 4–6 experiments. Data were statistically analyzed with one-way ANOVA followed by Tukey post hoc multiple comparison analysis between groups. Values that do not share the same letter are significantly different from each other (*p* ≤ 0.05).

We observed unique differences in the molecular weights of ACE2 across all three human primary endothelial cells studied. In HUVECs, we observed ACE2 at the predicted molecular weight of 90–97 kD which is deemed to be native ACE2 (Figure 3C). However, in HAECs and HMVECs, we observed ACE2 between 90–97 kD, as well as ACE2 fragments at ~52 kD (Figure 3A,E). Evidence exists for novel enzymatically active ACE2 fragments in renal tissue in vivo; however, to our knowledge, this is the first identification of these fragments in vitro [20]. Further, ACE2 was expressed as a prominent double band at the 90–97 kD range in HMVECs (Figure 3E) compared with the single bands observed in HAECs (Figure 3A) and HUVECs (Figure 3C). In HAECs, native ACE2 was significantly greater in male-derived cells under basal conditions compared with all other groups (Figure 3A,B), which paralleled the lower molecular weight ACE2 fragment (Figure 3A,G). In HUVECs, native ACE2 was not significantly different between male- and female-derived cells, while inflammatory insult with TNF-α reduced ACE2 protein expression irrespective of sex (Figure 3C,D). We did not detect the expression of ACE2 fragments in HUVECs (Figure 3C,H). In HMVECs, native ACE2 was similar to HAECs, in which male-derived cells under basal conditions were significantly greater expressed than all other groups (Figure 3E,F). However, reduction of ACE2 was more prominent in male cells compared with female cells, as TNF-α treatment did not significantly reduce ACE2 expression in female HMVECs. In contrast, the lower molecular weight ACE2 fragment was expressed similarly between male and female HMVECs, while inflammatory insult significantly reduced its expression irrespective of sex (Figure 3I,L). 

Cell line-specific differences in *AGT2R* mRNA expression were observed between sexes and treatment conditions. In HAECs, *AGT2R* was not different between sexes; however, inflammatory insult with TNF-α significantly reduced its expression in both sexes compared to basal conditions (Figure 3J). In HUVECs, *AGT2R* mRNA was significantly greater in female-derived cells under basal conditions compared to males irrespective of treatment, while TNF-α significantly reduced *AGT2R* mRNA in female-derived cells (Figure 3K). In contrast, *AGT2R* mRNA was significantly greater in male HMVECs compared to female-derived cells under both basal and inflammatory conditions (Figure 3L). MAS receptor (*MAS)1* mRNA expression was not different between sexes and treatments in HAECs (Figure 3M), while in HUVECs, *MAS1* mRNA was significantly greater in female-derived cells compared to male-derived cells irrespective of treatment (Figure 3N). In HMVECs, male-derived cells under basal conditions expressed greater *MAS1* mRNA than females irrespective of treatment and TNF-α treated males (Figure 3O). In short, our data on the expression of RAS components indicate that ACE1 does not differ between sexes within endothelial cell lines. However, ACE2 protein, *AGT1R*, *AGT2R* and *MAS1* gene expression are uniquely expressed in a sex-specific manner depending on cell origin and respond to inflammatory stimuli in a distinct manner.

### 3.3. NADPH-Oxidase (NOX) Expression

**Figure 4:**
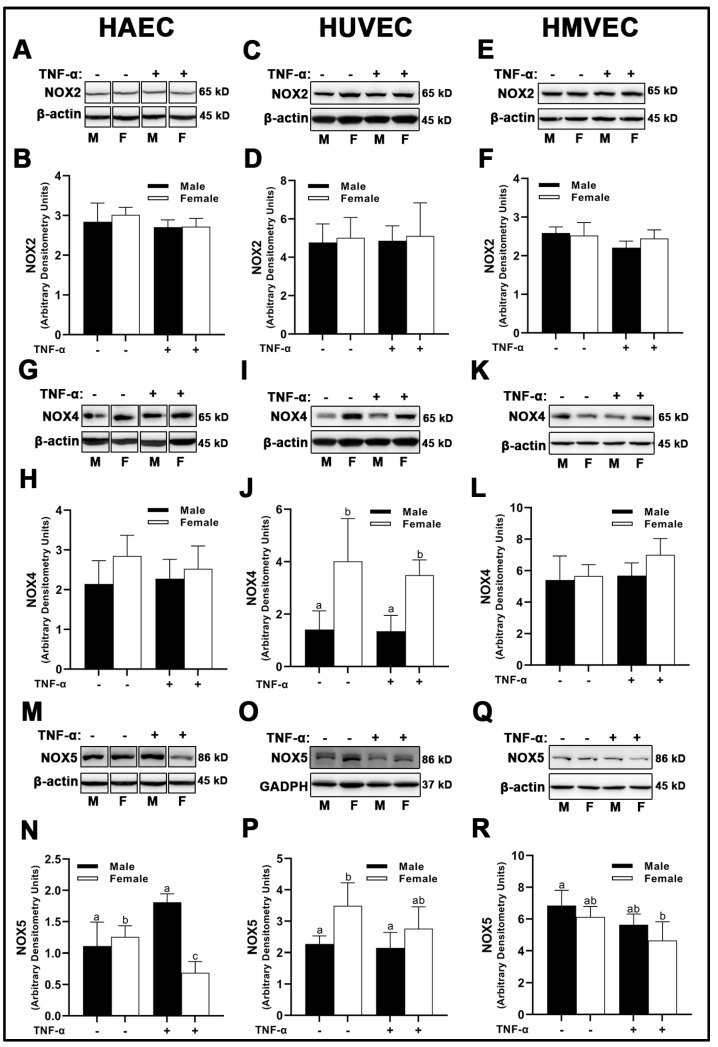
Expression of pro-oxidant NOX isoforms in male and female endothelial cells. HAECs, HUVECs and HMVECs were cultured in starvation medium at ~80% confluency for 24 h with or without TNF-α (20 ng/mL). Protein expression of NOX2 (**A**–**F**), NOX4 (**G**–**L**) and NOX5 (**M**–**R**) were determined by Western blot. Quantification was performed using Image Lab (Bio-Rad Laboratories, Inc.). Protein was normalized to respective control bands (β-actin or GAPDH). Data are expressed as mean ± SD from 4–6 experiments. Data were statistically analyzed with one-way ANOVA followed by Tukey post hoc multiple comparison analysis between groups. Values that do not share the same letter are significantly different from each other *(p* ≤ 0.05).

We observed no sex- or treatment-dependent differences in NOX2 protein expression in HAECs, HUVECs, or HMVECs (Figure 4A–E). With regard to NOX4, female HUVECs expressed greater NOX4 irrespective of TNF-α treatment (Figure 4I,J), while no differences were observed in HAECs (Figure 4G,H) and HMVECs (Figure 4K,L). NOX5 expressed uniquely in a sex- and treatment-dependent manner among the three human primary endothelial cells studied. In HAECs, NOX5 was not different between male and female-derived cells under basal conditions (Figure 4M,N); however, inflammatory insult increased NOX5 in male HAECs, while NOX5 was decreased in female cells, illustrating a clear divergence in the sex response. In HUVECs, male cells, irrespective of treatment, expressed lower NOX5 compared to female cells under basal conditions (Figure 4O,P). In HMVECs, TNF-α treatment reduced NOX5 expression in female cells compared to male basal cells Figure 4Q,R. In sum, our data denote that sex differences in NOX protein expression are dependent upon the isoform evaluated, with the greatest heterogeneity observed in NOX5 protein expression across all three human primary endothelial cells evaluated. 

### 3.4. Endogenous Antioxidant Expression

**Figure 5:**
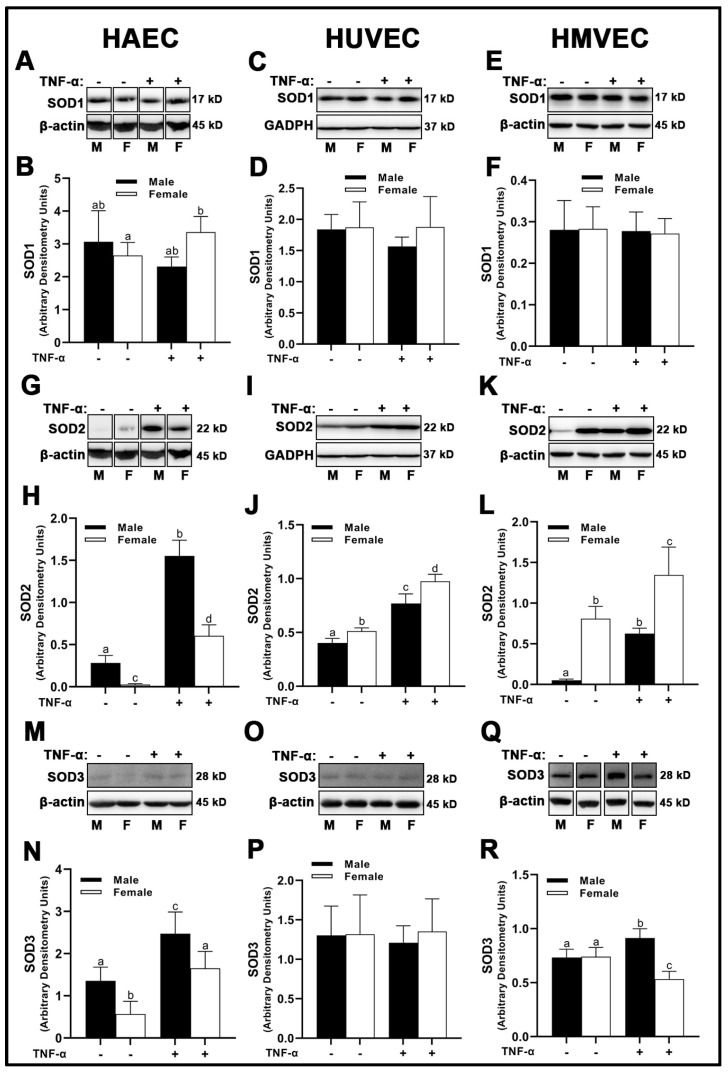
Expression of SOD isoforms in male and female endothelial cells. HAECs, HUVECs and HMVECs were cultured in starvation medium at ~80% confluency for 24 h with or without TNF-α (20 ng/mL). Protein expression of SOD1 (**A**–**F**), SOD2 (**G**–**L**) and SOD3 (**M**–**R**) were determined by Western blot. Quantification was performed using Image Lab (Bio-Rad Laboratories, Inc.). Protein was normalized to respective control bands (β-actin or GAPDH). Data are expressed as mean ± SD from 4–6 experiments. Data were statistically analyzed with one-way ANOVA followed by Tukey post hoc multiple comparison analysis between groups. Values that do not share the same letter are significantly different from each other (*p* ≤ 0.05).

In HAECs, we observed no sex differences in superoxide dismutase (SOD)1 expression under basal conditions (Figure 5A,B); however, inflammatory insult with TNF-α reduced SOD1 in male- compared with female-derived HAECs. HUVECs and HMVECs did not display any sex or treatment differences with respect to SOD1 (Figure 5C–F). SOD2 protein expression was different among all three human primary endothelial cells assessed in a sex- and treatment-dependent manner. In HAECs, SOD2 was greater in male cells (Figure 5G,H), while in HMVECs and HUVECs, the inverse was observed (Figure 5I–L). Further, while SOD2 was increased with TNF-α treatment in all cells, the magnitude of change was uniquely different. For example, in HAECs, TNF-α treatment resulted in a ~5.5- and ~23-fold increase in SOD2 expression in male- and female-derived cells, respectively (Figure 5G,H). In contrast, HUVEC male- and female-derived cells expressed a similar ~1.9-fold increase in SOD2 expression with TNF-α treatment (Figure 5I,J). HMVECs increased SOD2 expression to a greater degree in male-derived cells (~12-fold increase) compared to female HMVECs (~1.6-fold increase) illustrating clear sex- and cell-dependent differences in mitochondrial sensitivity to inflammatory stimuli (Figure 5K,L). In HAECs, SOD3 reflected a similar pattern to SOD2 data, with male HAECs expressing greater SOD3 compared to females under basal and inflammatory conditions (Figure 5M,N). However, HUVEC SOD3 protein expression was not significantly different between sexes or treatments (Figure 5O,P). In contrast, while HMVEC male and female-derived cells expressed similar concentrations of SOD3 under basal condition, inflammatory insult significantly increased male SOD3, while female SOD3 significantly decreased compared to other groups (Figure 5Q,R). 

**Figure 6:**
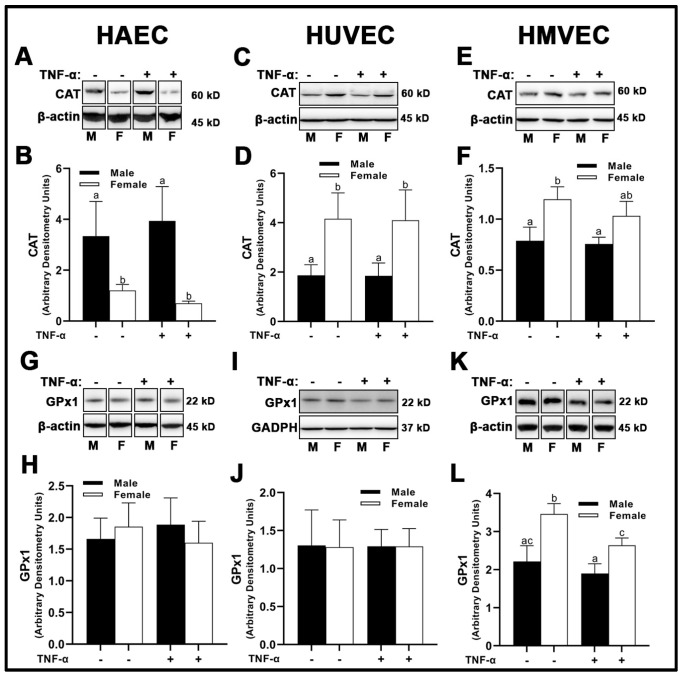
Expression of peroxidases in male and female endothelial cells. HAECs, HUVECs and HMVECs were cultured in starvation medium at ~80% confluency for 24 h with or without TNF-α (20 ng/mL). Protein expression of CAT (**A**–**F**) and GPx1 (**G**–**L**) were determined by Western blot. Quantification was performed using Image Lab (Bio-Rad Laboratories, Inc.). Protein was normalized to respective control bands (β-actin or GAPDH). Data are expressed as mean ± SD from 4–6 experiments. Data were statistically analyzed with one-way ANOVA followed by Tukey post hoc multiple comparison analysis between groups. Values that do not share the same letter are significantly different from each other (*p* ≤ 0.05).

With respect to the peroxidase catalase (CAT), there were no treatment effects across all three human primary endothelial cells evaluated (Figure 6A–F); however, sex differences were apparent. Male HAECs expressed greater CAT than females (Figure 5A,B), while the inverse was observed in HUVECs (Figure 6C,D) and HMVECs (Figure 6E,F). Interestingly, glutathione peroxidase (GPx)1 was unchanged irrespective of sex or treatment in HAECs (Figure 6G,H) and HUVECs (Figure 6I,J), while HMVECs expressed reduced GPx1 protein with TNF-α treatment with no observed sex-differences (Figure 6K,L). Thus, these data illustrate a complete lack of uniformity in antioxidant protein expression with respect to sex and inflammatory insult across all three human primary endothelial cells. In summary, our data illustrate that sex alone does not predict antioxidant enzyme expression in a uniform manner in endothelial cells, as each human primary endothelial cell evaluated expressed SOD1-3, CAT and GPx1 in a sex-specific manner which differed based on cell origin. Further, we show that SOD2 is particularly sensitive to inflammatory insult (TNF-α), and its expression is universally increased relative to basal conditions independent of sex.

**Figure 7:**
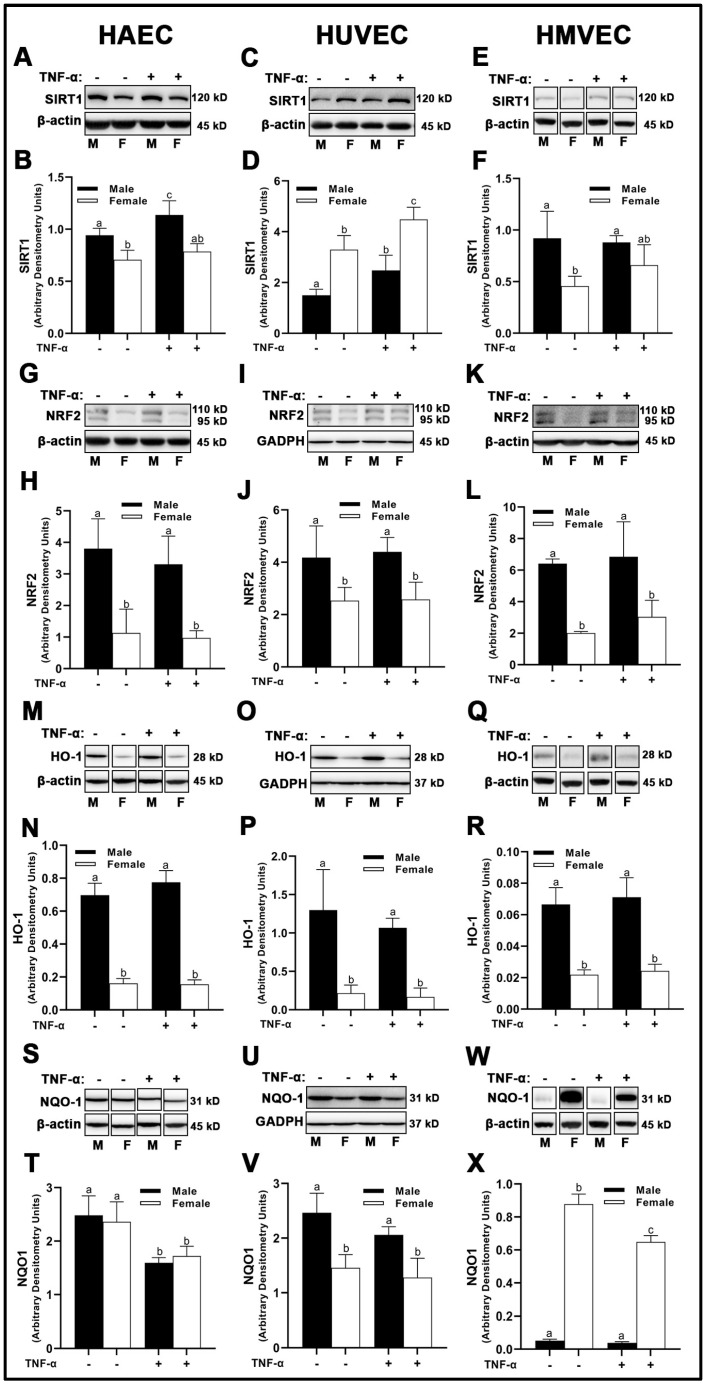
Expression of endogenous antioxidant regulators in male and female endothelial cells. HAECs, HUVECs and HMVECs were cultured in starvation medium at ~80% confluency for 24 h with or without TNF-α (20 ng/mL). Protein expression of SIRT1 (**A**–**F**), NRF2 (**G**–**L**) and HO-1 (**M**–**R**) and NQO1 (**S**–**X**) were determined by Western blot. Quantification was performed using Image Lab (Bio-Rad Laboratories, Inc.). Protein was normalized to respective control bands (β-actin or GAPDH). Data are expressed as mean ± SD from 4–6 experiments. Data were statistically analyzed with one-way ANOVA followed by Tukey post hoc multiple comparison analysis between groups. Values that do not share the same letter are significantly different from each other (*p* ≤ 0.05).

In HAECs, sirtuin (SIRT)1 protein expression was greater in males compared to females (Figure 7A,B), while TNF-α treatment enhanced SIRT1 expression in males, but not females. Similar results were obtained in HMVECs (Figure 7E,F), although TNF-α was less effective in increasing SIRT1 expression. Inverse findings were observed in HUVECs, as female-derived cells expressed greater SIRT1 compared to male HUVECs, and TNF-α treatment significantly increased SIRT1 expression in both sexes (Figure 7C,D). 

Total nuclear factor erythroid 2–related factor (NRF)2 and heme oxygenase (HO)-1 protein expression was higher in male-derived cells compared with female-derived cells across all endothelial cells evaluated with no effects of inflammatory insult (Figure 7G–R), suggesting increased antioxidant response element (ARE) activity in males. However, NADPH quinone dehydrogenase (NQO1) expressed uniquely in HAECs, HUVECs, and HMVECs. In HAECs, we observed no sex differences in NQO1 expression (Figure 7S,T), while TNF-α significantly reduced NQO1 in both sexes. In contrast, clear sex differences were noted in HUVECs and HMVECs. Male-derived HUVECs expressed higher NQO1 compared to female HUVECs (Figure 7U,V), while TNF-α treatment did not significantly reduce NQO1 expression between sexes. In HMVECs, inverse expression of NQO1 between sexes was observed with a larger magnitude of difference, as NQO1 was very weakly expressed in males compared to females (~17-fold difference) (Figure 7W,X). TNF-α reduced NQO1 in female HMVECs, but not in male HMVECs. In brief, our data indicate that NRF2 and HO-1 are uniquely increased in male-derived cells irrespective of cell origin and are not influenced by inflammatory stimulation with TNF-α. However, SIRT1 and NQO1 were distinctly expressed between sexes among differing all three human primary endothelial cells studied.

### 3.5. Cellular Reactive Oxygen Species

**Figure 8:**
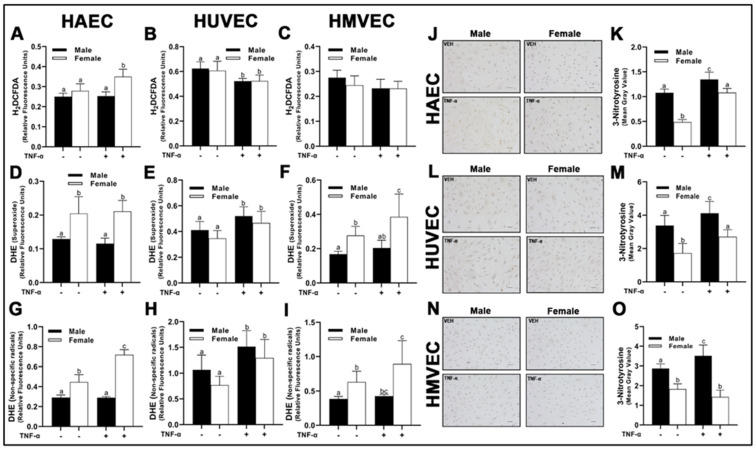
Redox activity in male and female endothelial cells. HAECs, HUVECs and HMVECs were cultured at ~80% confluency for 24 h in starvation medium with or without TNF-α (20 ng/mL). H_2_DCFDA or DHE dissolved in dimethyl sulfoxide were added to wells (10 µM final concentration) followed by 30 min incubation (37 °C and 5% CO_2_). Phenol red-free starvation medium supplemented with NucBlue™ (1 drop/mL) was added. Fluorescence was read at the following Ex/Em: 495/527 (**A**–**C**; H_2_DCFDA), 518/606 (**D**–**F**; DHE; O_2_^−^) 480/576 (**G**–**I**; DHE; non-specific radicals) and 360/460 (Hoechst 33342 with NucBlue™). Fluorometric values for H_2_DCFDA and DHE were normalized to Hoechst 33342. Cells were also fixed on poly-L-lysine cover slips and stained with DAB diluent following incubation with nitrotyrosine primary antibody (**J**–**O**). Quantification was performed with Image J. The scale bar is 50 µm and objective magnification is 20×. Data are expressed as mean ± SD from 3–6 experiments. Data were statistically analyzed with one-way ANOVA followed by Tukey post hoc multiple comparison analysis between groups. Values that do not share the same letter are significantly different from each other (*p* ≤ 0.05).

To evaluate the net impact of these differences in pro-oxidant and antioxidant enzyme expression, we fluorometrically assessed intracellular O_2_^−^ with dihydroethidium (DHE), as well as H_2_O_2_ with 2′,7′-dichlorodihydrofluorescein diacetate (H_2_DCFDA) (Figure 7). DHE however is not a O_2_^−^-specific probe [21], thus we assessed other non-specific radicals (hydroxyl radical, peroxyl radicals, glutathionyl radicals, etc.) at an additional Ex/Em more appropriate for their detection (480/576 nm). In HAECs probed with H_2_DCFDA, we did not observe basal sex differences (Figure 8A); however, inflammatory insult with TNF-α resulted in a significant increase in fluorometric intensity in female-derived cells compared with basal females and males irrespective of treatment. In contrast, intracellular H_2_O_2_ in HUVECs was significantly lower in cells treated with TNF-α (Figure 8B), irrespective of sex with no basal sex differences, while no differences in H_2_O_2_ expression were detected in HMVECs (Figure 8C). 

In HAECs, O_2_^−^ concentrations were greater in female-derived cells compared to males HAECs (Figure 8D), which reflected concentrations of non-specific radicals (Figure 8G). Interestingly, female HAECs treated with TNF-α had a significantly greater concentration of non-specific radicals compared to basal female cells, while male cells treated with TNF-α did not have any response to treatment. In HUVECs treated with DHE, both sexes responded to TNF-α, with a significant rise in fluorescent intensity in both Ex/Em wavelengths compared to basal cells (Figure 8E,H), although no significant sex-differences were observed. In contrast to HUVECs, HMVECs expressed similarly to HAECs, in which female-derived cells, irrespective of treatment, had greater concentrations of O_2_^–^ and non-specific radicals (Figure 8F,I). However, female-derived HMVECs treated with TNF-α had increased fluorescent intensity, while male-derived cells did not.

We next evaluated oxidation of NO via immunocytochemistry by measuring nitrotyrosine, another indicator of oxidative stress. In contrast to DHE data above, all three human primary endothelial cells expressed greater nitrotyrosine in male-derived cells compared to female-derived cells irrespective of treatment (Figure 8J–O). We also observed a consistent increase in nitrotyrosine staining following TNF-α treatment, although these effects were not apparent in HMVECs. In summary, our data illustrate unique sex-specific release of H_2_O_2_ and O_2_^−^ which was dependent upon endothelial cell origin. However, nitrotyrosine levels were higher in males irrespective of endothelial cell origin.

### 3.6. Endothelial Nitric Oxide Synthase (eNOS) and Nitric Oxide (NO) Bioavailability

**Figure 9:**
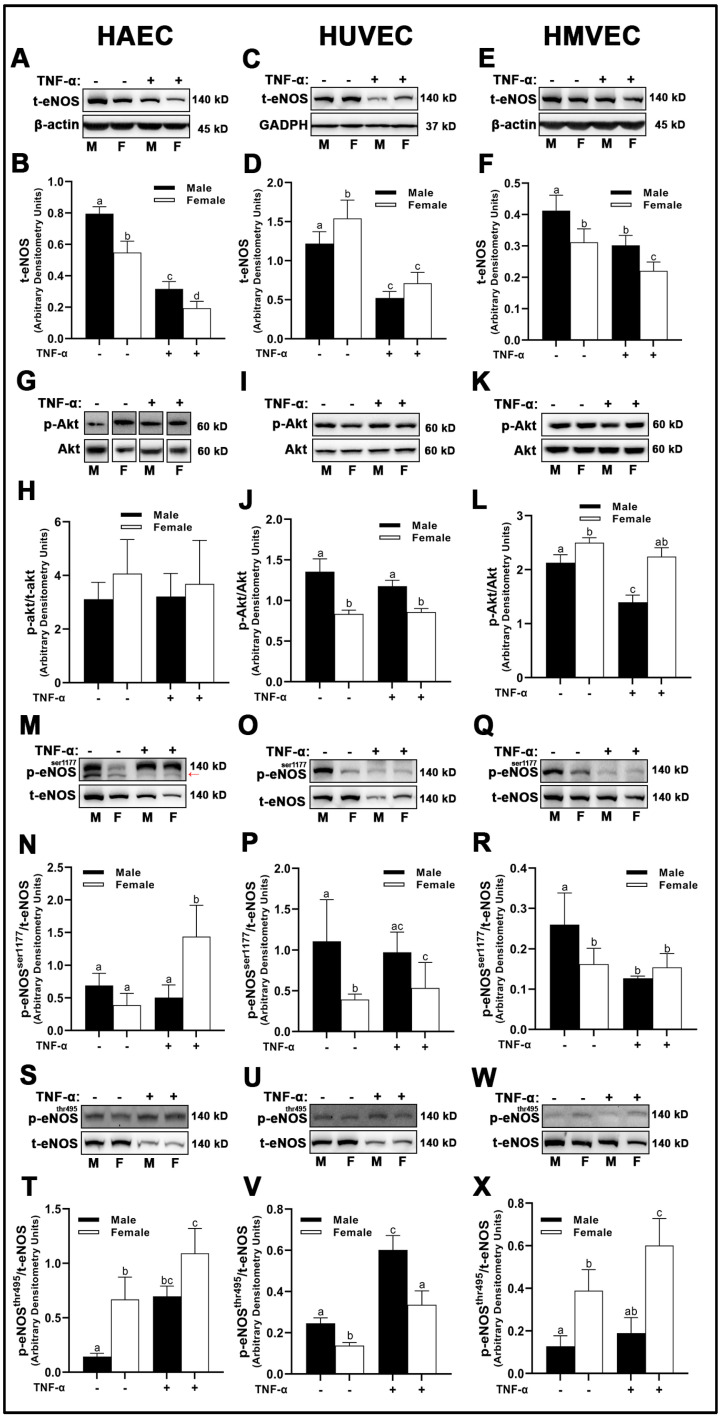
Regulation of eNOS in male and female endothelial cells. HAECs, HUVECs and HMVECs were cultured in starvation medium at ~80% confluency for 24 h with or without TNF-α (20 ng/mL). Protein expression of t-eNOS (**A**–**F**), p-akt (**G**–**L**), p-eNOS^Ser1177^ (**M**–**R**) and p-eNOS^Thr495^ (**S**–**X**) were determined by Western blot. Quantification was performed using Image Lab (Bio-Rad Laboratories, Inc.). Band expression of t-eNOS and t-akt were normalized to respective control bands (β-actin or GAPDH). For phosphorylated proteins that were not probed on the same membrane as their respective total proteins, they were first normalized to control proteins (β-actin or GAPDH) followed by normalization with their respective normalized total proteins from a separate membrane. Data are expressed as mean ± SD from 4–6 experiments. Data were statistically analyzed with one-way ANOVA followed by Tukey post hoc multiple comparison analysis between groups. Values that do not share the same letter are significantly different from each other (*p* ≤ 0.05).

In both male-derived HAECs and HMVECs, eNOS protein expression was significantly greater compared to female-derived cells (Figure 9A,B,E,F). In HUVECs, inverse sex differences were observed, as female-derived cells expressed significantly greater eNOS than male-derived cells (Figure 9C,D). We did not observe any sex or treatment differences in Akt phosphorylation in HAECs (Figure 9G,H). However, male-derived HUVECs had greater Akt phosphorylation compared with female-derived cells (Figure 9I,J), while the inverse was true in HMVECs (Figure 9K,L). Further, we observed that TNF-α treatment in HMVECs significantly reduced Akt phosphorylation in male-derived cells compared with basal conditions, but this effect did not occur in female-derived cells. With respect to phosphorylation of eNOS at Ser^1177^, we observed a complete lack of uniformity between sexes and cell types. In HAECs, Ser^1177^ phosphorylation increased significantly in female-derived cells treated with TNF-α compared with basal cells of both sexes and TNF-α-treated males, with no other differences between groups detected (Figure 9M,N). In HUVECs, male-derived cells expressed greater Ser^1177^ phosphorylation of eNOS compared with female-derived cells, with no significant TNF-α effects (Figure 9O,P). In contrast, male-derived HMVECs expressed greater phosphorylation of Ser^1177^ in basal male-derived cells compared with all other groups with no other significant differences detected (Figure 9Q,R). In both HAECs and HMVECs, phosphorylation of eNOS at Thr^495^ was significantly lower in male-derived compared with female-derived cells (Figure 9S,T,W,X), while the inverse was observed in HUVECs (Figure 9U,V). 

**Figure 10:**
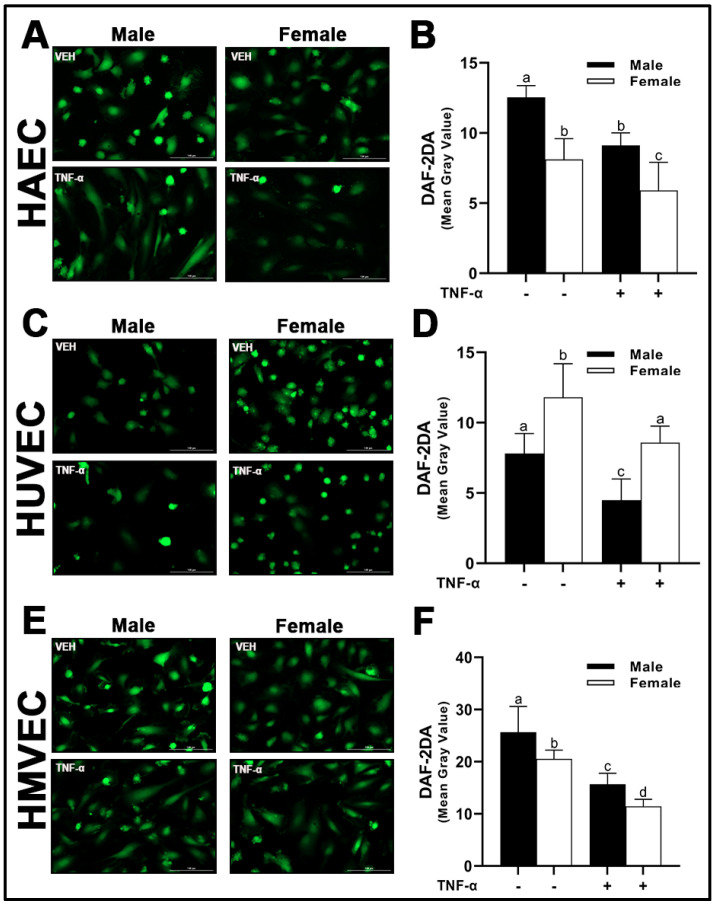
Nitric oxide bioavailability in male and female endothelial cells. HAECs, HUVECs and HMVECs were cultured in starvation medium at ~80% confluency for 24 h with or without TNF-α (20 ng/mL). DAF-2DA was detected by fluorescence microscopy (**A**,**C**,**E**) at 485/528 (Ex/Em). Fluorescent intensity of cells was quantified with Image J (**B**,**D**,**F**). Data are expressed as mean ± SD from 4–6 experiments. Data were statistically analyzed with one-way ANOVA followed by Tukey post hoc multiple comparison analysis between groups. Values that do not share the same letter are significantly different from each other (*p* ≤ 0.05).

Following the assessment of eNOS protein expression and its phosphorylation sites, intracellular NO concentrations were then assessed by diaminofluorescein-2 diacetate (DAF-2 DA), a cell-permeable molecular probe, which is deacetylated by intracellular esterases to DAF-2 and reacts with NO to form the highly fluorescent compound, triazolofluorescein [22]. As expected, we observed a reduction in intracellular NO with TNF-α treatment across all three human primary endothelial cells studied irrespective of sex. In both HAECs and HMVECs, we observed significantly greater intracellular NO in male-derived cells compared with female-derived cells (Figure 10A,B,E,F). In contrast, a greater concentration of NO in female-derived HUVECs was observed compared to male-derived cells (Figure 10C,D). Briefly, our data demonstrate that eNOS protein expression and phosphorylation status as well as NO release differ between sexes and this difference is dependent upon endothelial cell origin. We further illustrate that phosphorylation of eNOS at Ser^1177^ does not require relative increases in Akt phosphorylation between sexes.

### 3.7. Inflammation

**Figure 11:**
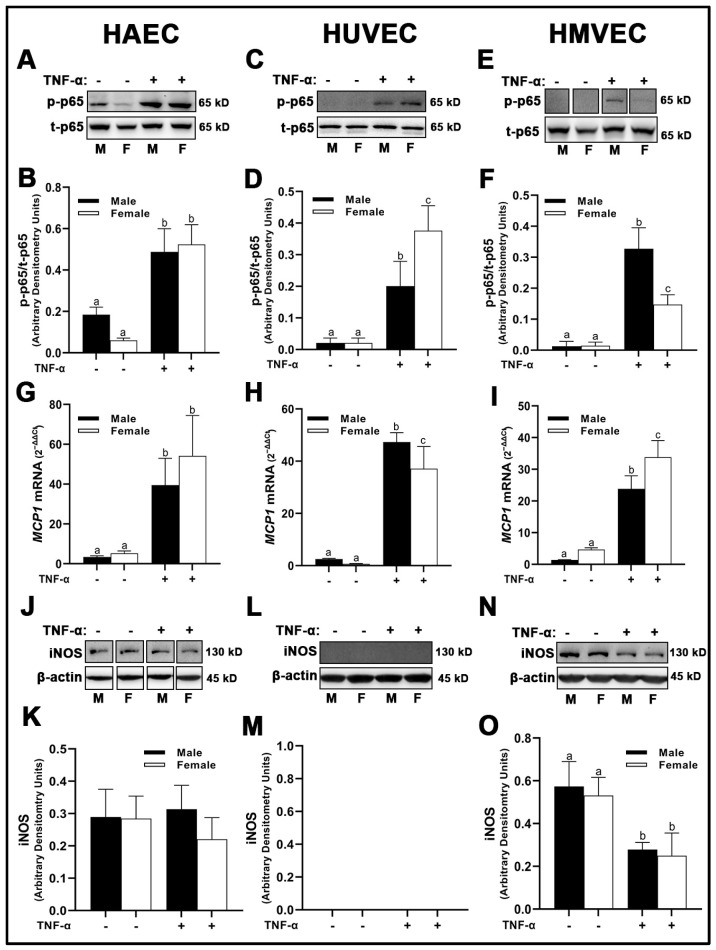
Expression of inflammatory markers in male and female endothelial cells. HAECs, HUVECs and HMVECs were cultured in starvation medium at ~80% confluency for 24 h with or without TNF-α (20 ng/mL). Protein expression of phospho-p65 (**A**–**F**) and iNOS (**J**–**O**) were determined by Western blot. Quantification was performed using Image Lab (Bio-Rad Laboratories, Inc.). Protein was normalized to respective control bands (β-actin). Phosphorylated proteins were normalized to respective total proteins. Gene expression of MCP-1 (**G**–**I**) were determined with qPCR. Genes of interest were normalized to housekeeping gene cyclophilin. Data are expressed as mean ± SD from 4–6 experiments. Data were statistically analyzed with one-way ANOVA followed by Tukey post hoc multiple comparison analysis between groups. Values that do not share the same letter are significantly different from each other (*p* ≤ 0.05).

In HAECs, we observed basal sex differences in phospho-NF-κB p65 subunit protein expression (Figure 11A,B), with male-derived cells having greater expression compared to female-derived cells. While TNF-α increased HAEC NF-κB phosphorylation of p65 subunit, no sex differences were observed. In contrast, basal differences in phospho-NF-κB p65 subunit expression in HUVECs (Figure 11C,D) and HMVECs (Figure 11E,F) were not detected between sexes, while following TNF-α, phospho-NF-κB p65 subunit was increased in female- compared to male-derived HUVECs, and the opposite was observed in HMVECs. While no basal sex differences were observed, *MCP1* gene expression (Figure 11G–I), as expected, was increased following TNF-α treatment in all three human primary endothelial cells evaluated. *MCP1* mRNA was greater in male-derived HUVECs (Figure 11H) compared with female-derived HUVECs, and the opposite was observed in HMVECs (Figure 11I) with no differences between male- and female-derived cells in HAECs (Figure 11G). With regard to inducible NO synthase (iNOS), we observed cell origin-specific differences but no sex differences under either basal or TNF-α conditions. In HAECs, expression of iNOS was not changed by treatment with TNF-α (Figure 11J,K) while in HMVECs, iNOS protein was reduced with TNF-α treatment (Figure 11N,O). In HUVECs, iNOS protein could not be detected by Western blot even in trace amounts following extended chemiluminescent exposure (data not shown) (Figure 11L,M), suggesting that iNOS protein may not play a substantial role in HUVECs compared to HAECs and HMVECs. In short, our data indicate that protein phosphorylation of NF-κB p65 subunit, gene expression of *MCP1* and iNOS protein expression are distinct between sexes under both basal and inflammatory conditions, and that these differences depend upon endothelial cell origin.

## 4. Discussion

The purpose of this study was to investigate whether in vitro mechanisms of endothelial function differ based on cell origin and/or biological sex. We observed intrinsic differences in expression of sex receptors, RAS elements, redox enzymes and redox status, transcriptional regulators of antioxidant enzymes, eNOS expression, NO bioavailability as well as inflammatory expression among all three human primary endothelial cells studied and between sexes under both basal and inflammatory conditions. Here, we show that (1) sex differences exist within commonly utilized human primary endothelial cells, (2) sex differences are unique between endothelial cells derived from different tissues, and (3) endothelial cells behave uniquely to inflammatory insult in a sex- and cell origin-dependent manner. Thus, our data suggest that both cell origin and biological sex are important considerations from both a methodological and translational perspective 

A prior study by Cattaneo et al. [23] investigated cellular mechanisms underlying sex differences in CVD. HUVECs from fraternal twins in both cell culture and freshly flushed from umbilical veins revealed that female-derived cells expressed greater eNOS and NO compared with male-derived cells. These results support our findings with respect to HUVECs. However, our findings also suggest data from HUVECs may not translate to cells from other tissues such as aorta and dermal microvasculature. To appropriately study these in vitro models, endothelial cells from the tissue of interest should be utilized with considerations for sex.

**Figure 12:**
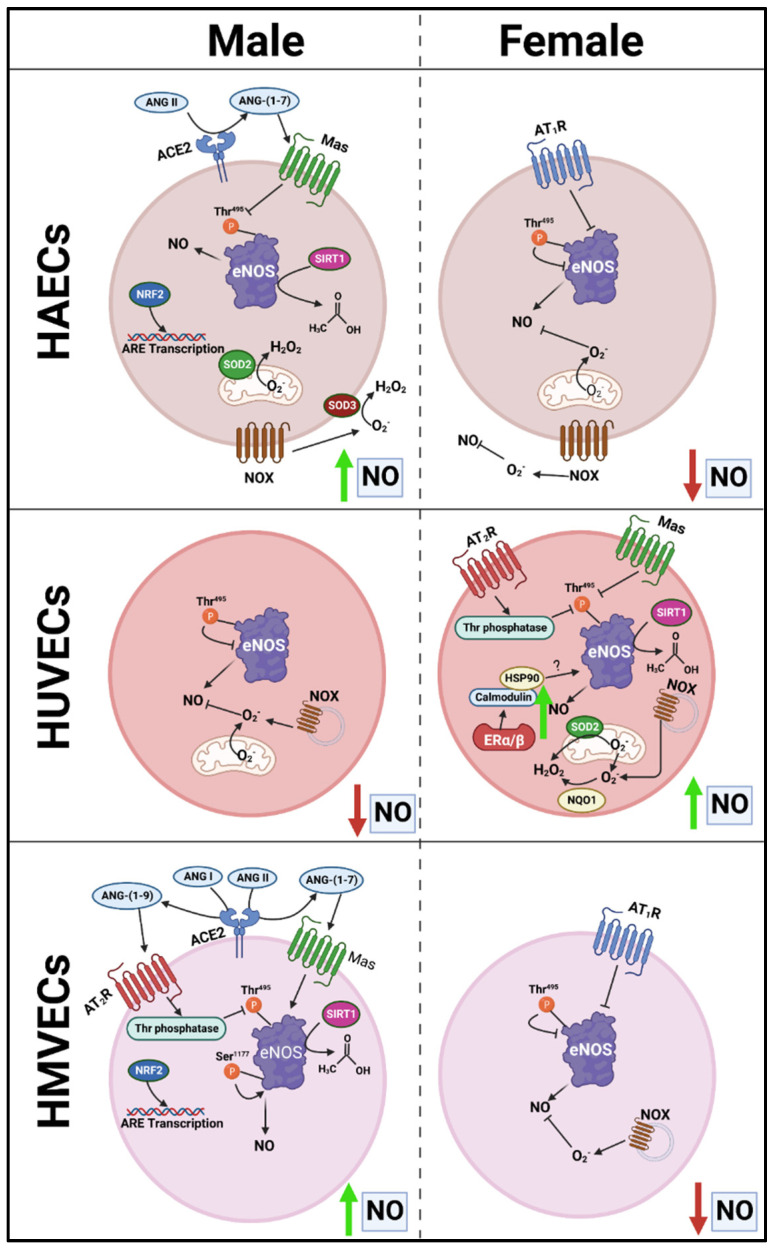
Proposed schematic presentation of sex-specific differences in protein expression between endothelial cells that may mediate sex- and cell-specific discrepancies in NO bioavailability.

With respect to NO bioavailability in the present investigation, we observed a consistent association between total eNOS protein and NO, as well as an inverse association between eNOS^Thr495^ phosphorylation and NO. However, the pathways responsible for NO maintenance are likely far more complex than merely total protein and Thr^495^ phosphorylation status, as numerous differences in the pathways involved in the regulation of NO were found between cells (Figure 12). Additionally, the pathways described in Figure 12 that may hypothetically regulate NO may not be involved to the same extent in endothelial cells derived from different tissues due to the complex molecular environment. We aimed to capture this in the figure; however, it would be difficult to fully characterize differences in the regulation of NO among these three types of human primary endothelial cells due to inconsistencies observed in the expression of specific molecular pathways known to influence eNOS in these cells. For example, AT_1_R can form a complex with eNOS, reducing its activity [24], and indeed, we observed that *AGT1R* mRNA expression between sexes within HAECs and HMVECs (Figure 2) was inversely related to NO synthesis (Figure 10). In HUVECs specifically, our data suggest AT_1_R was not a plausible mechanism to explain sex differences in NO. However, *AGT2R* mRNA expression was increased in female derived HUVECs with respect to males which was related to differences in NO and these findings were also observed in HMVECs, but not in HAECs (Figure 3). Additionally, ERα was increased in female derived HUVECs (Figure 1). Increased ERα expression can directly increase eNOS activity via increased Ca^2+^ flux [25,26], driving calmodulin activity leading to increased heat shock protein (HSP)90/calmodulin-eNOS protein interaction [27]. In HAECs, ERα was not significantly different between sexes, and HMVEC ERα expression was increased in females suggesting that this was not the driving factor in NO synthesis in these cells since NO was higher in males. Despite these differences, across all three human primary endothelial cell origins, ROS production, particularly O_2_^–^ production and other oxidation products as assessed by DHE were inversely related to NO synthesis (Figure 8 and Figure 10), suggesting that redox status is a significant contributor to bioavailable NO. 

The source of ROS generation in the three types of human primary endothelial cells used in the present investigation cannot be explained by differences in NOX2, NOX4, and NOX5 protein expression alone (Figure 4). SOD2, a mitochondrial dismutase, was significantly increased under inflammatory conditions, suggesting that mitochondrial-derived ROS may be the major source of ROS in endothelial cells (Figure 5). Indeed, Corda et al. [28] has previously reported that HUVECs treated with TNF-α rapidly produce mitochondrial-derived ROS, and that inhibitors of NOX and xanthine oxidase did not reduce ROS production. In the present investigation, we found no consistent relationship between antioxidant enzyme expression and NO bioavailability, suggesting unique ROS-neutralizing antioxidant enzyme profiles between cells responsible for these redox differences. This may explain why nitrotyrosine was expressed to a greater extent in males across all three human primary endothelial cells despite the mismatch with DHE fluorescence in HAECs and HMVECs (Figure 8). However, we did find that, between sexes, under basal conditions, SIRT1 was associated with NO (Figure 7). This may be due to increased eNOS catalytic activity following deacetylation by SIRT1 [29]. Notably, SIRT1 increased with TNF-α stimulation despite reduced NO, suggesting that the molecular activity of SIRT1 shifted, as evidenced by increased SOD2 synthesis. 

With respect to NRF2, NQO1 is classically considered a transcriptional product of NRF2; however, this was not observed in the present study (Figure 7), and Jun family proteins are required in coordination with NRF2 for NQO1 synthesis possibly explaining this discrepancy [30]. This contrasted with HO-1 which closely tied with NRF2 expression in all three types of human primary endothelial cells studied here. Interestingly, males expressed greater NRF2. NQO1 appears localized to mitotic spindles in a number of human cell lines [31], suggesting that NQO1 plays a role in protecting the cell from DNA-mediated oxidative damage during mitosis. The fact that NQO1 was nearly absent in male-derived HMVECs, while substantially increased in female-derived HMVECs with the inverse observed with HO-1 expression is of peculiarity. This sex-difference was not observed in HAECs and HUVECs. The consequences of these sex differences in vivo in the context of CVD are unclear. However, males tend to have an earlier onset of diabetic neuropathy compared to females [32], suggesting increased dysregulation of microvascular endothelial cells in males compared to females possibly due to reduced NQO1, although this is speculation and requires further investigation.

Two additional important findings in this investigation were (1) that NF-κB p65 subunit phosphorylation was not related to cellular redox status between sexes within treatment groups and (2) NF-κB p65 subunit phosphorylation did not reflect sex differences in *MCP1* mRNA expression (Figure 11). ROS are known to react with reactive cysteine residues on IκB kinase (IKK) [33], leading to the downstream phosphorylation and release of the p65 subunit of the NF-κB complex, leading to p65 translocation to the nucleus and inflammatory cytokine transcription. However, IKK is also regulated by the parallel inflammatory pathways involved in MAPK signaling. The MAPK kinase kinase transforming growth factor-β (TGF-β)-activated kinase 1 can also phosphorylate IKK [34], leading to p65 phosphorylation. With respect to the mismatch between *MCP1* mRNA and p65 subunit phosphorylation between sexes, it is likely that activator protein (AP)-1 is cooperatively involved in gene regulation of MCP-1, as AP-1 binding was identified in the promoter region of MCP-1 [35]. AP-1 is primarily regulated by MAPKs: p38MAPK and Jun amino-terminal kinase (JNK) [36], and considering the differential expression of proteins between sexes observed in the present study, there are likely sex differences in the MAPK pathway as well, although this was not explored.

### Limitations

The present investigation has several limitations that should be noted. Firstly, we do not have multiple donors for each endothelial cell type to assess as a representative group, and it is possible there may be interindividual variation between donors which would not be detected by using cells from a single donor. However, our eNOS and NO results are similar to prior reports on HUVECs [23], suggesting our data are representative of commercially purchased endothelial cells. Regardless, future investigations should utilize multiple donors to identify if interindividual differences exist among healthy, age-matched subjects. Second, while all cell donors were considered healthy, we do not have a complete medical history of the individuals, and it is possible that there may be unstated health conditions that may affect some of the pathways studied. Third, although we did a thorough assessment of common pathways implicated in endothelial dysfunction, we did not investigate all related pathways. Fourth, endothelial cells may behave differently in vitro than in their native state in vivo where the smooth muscle and innervation are intact, and cells are subjected to hormones and other growth factors produced by the body. Lastly, we assessed gene expression of AT_1_R, AT_2_R and Mas1 rather than their protein expression. While genes may not always reflect protein expression, it is important to note that commercial antibodies to detect G-protein coupled receptors are generally non-specific [37,38,39]. Thus, it would be less desirable to use commercial antibodies to detect these proteins.

## 5. Conclusions

In summary, our findings illustrate that endothelial cells derived from one tissue and one sex are not molecularly homogenous and are not interchangeable. Investigators should carefully consider the sex and endothelial cell derivation when studying vascular biology. These considerations extend beyond endothelial cells, with likely cell- and sex-specific differences in other human primary cells which are traditionally used interchangeably, such as epithelial cells of the airways (bronchial, small airway, tracheal, etc.), vascular smooth muscle cells (coronary artery, aortic, pulmonary, etc.) and renal epithelial cells (proximal tubule and cortical). Intrinsic differences exist among distinct human primary endothelial cells with potential sex-differences, which may limit extrapolation to the endothelium as a whole.

## Data Availability

Data are contained within the article.

## Supplementary Materials

The following supporting information can be downloaded at: https://www.mdpi.com/article/10.3390/cells12010093/s1, Table S1: Reagents; Table S2: Primer Sequences.
