# Supplementary material for: Tissue Derivation and Biological Sex Uniquely Mediate Endothelial Cell Protein Expression, Redox Status, and Nitric Oxide Synthesis
Source: Cells. 2022 Dec 26;12(1):93. doi: 10.3390/cells12010093 (PMC9818567; doi:10.3390/cells12010093)
Supplement: Supplementary file 1 [file cells-12-00093-s001.zip › cells-2118953-supplementary.pdf]

Table S1. Reagents.

Reagents	Company	Catalog Number
anti-ACE1	Abcam	ab216476
anti-ACE1	Abcam	ab15348
anti-phospho-Akt	Cell Signaling Technologies	4060
anti-Akt	Cell Signaling Technologies	2920
anti-AR	Cell Signaling Technologies	5153
anti-β-actin	Cell Signaling Technologies	3700
anti-catalase	Cell Signaling Technologies	14097
anti-eNOS	Cell Signaling Technologies	32027
anti-phospho-eNOS^{Ser1177}	Cell Signaling Technologies	9570
anti-phospho-eNOS^{Thr495}	Cell Signaling Technologies	9574
anti-ERα	Cell Signaling Technologies	8644
anti-ERβ	R&D Systems	PP-PPZ0506-00
anti-GAPDH	Cell Signaling Technologies	97166
anti-GPx1	Cell Signaling Technologies	3206
anti-HO-1	Cell Signaling Technologies	3683
anti-iNOS	Cell Signaling Technologies	20609
anti-phospho-NF-κB p65	Cell Signaling Technologies	3033
anti-NF-κB p65	Cell Signaling Technologies	8242
anti-nitrotyrosine	Abcam	ab7048
anti-NOX2	Abcam	ab180642
anti-NOX4	Abcam	ab131088
anti-NOX5	Abcam	ab191010
anti-NQO1	R&D Systems	AF7567
anti-NRF2	Novus Biologicals	NBP1-32822
anti-SIRT1	Cell Signaling Technologies	8469
anti-SOD1	Cell Signaling Technologies	2770
anti-SOD2	Cell Signaling Technologies	13141
anti-SOD3	Santa Cruz Biotechnology	Sc-271170
DAF-2 DA	Millipore-Sigma	251505-M
dNTP mix	Promega	U151B
EC Basal Medium	Cell Applications	211-500
Goat secondary	R&D Systems	HAF017
HAECs	Cell Applications	304-05a
HUVECs	Cell Applications	200-05n
HMVECs	Cell Applications	100-05a
HRP Substrate	EMD Millipore	WBLUF0500
ML-090	Cayman Chemical	15172
MLV RT 5X	Promega	M531A
M-MLV RT	Promega	M170B
Mouse secondary	Cell Signaling Technologies	7076S
MVEC Growth Medium	Cell Applications	112-500
Rabbit secondary	Cell Signaling Technologies	7074
RIPA lysis buffer	EMD Millipore	20-188
RNase inhibitor	Thermo Fisher Scientific	N8080119

SYBR green	BioRad	1725124
TNF-α protein, human	R&D Systems	210-TA
TRI Reagent	Sigma-Aldrich	T9424

Table S2. Primer Sequences

Gene	**Forward Sequence**	**Reverse Sequence**
NR3C4	5′-CCTGGCTTCCGCAACTTACA-3′	5′-GGACTTGTGCATGCGGTACTC-3′
AGT1R	5′- GCTGGCCCTTTGGCAATTAC-3	5′- GCTTCTTGGTGGATGAGCTT-3′
AGT2R	5'-TGCTATTACGTCCCAGCGTC-3′	5'-AGTGCCTAAACACACTCCTTCA-3'
MAS1	5′-GCTACAACACGGGCCTCTATCTG-3′	5′-TACTCCATGGTGGTCACCAAGC-3
MCP1	5′-GATCTCAGTGCAGAGGCTCG-3′	5′-TTTGCTTGTCCAGGTGGTCC-3′
CYP	5′-CTTCGAGCTGTTTGCAGACAA-3′	5′-AGATGCCAGCTGTATGCT-3′
